# Management strategy for facial arteriovenous malformations

**DOI:** 10.4103/0970-0358.44943

**Published:** 2008

**Authors:** P. S. Bhandari, L. P. Sadhotra, P. Bhargava, A. S. Bath, M. K. Mukherjee, Sanjay Maurya

**Affiliations:** Department of Plastic Surgery, Armed Forces Medical College and Command Hospital (SC) Pune-40, India

**Keywords:** Arteriovenous malformation, super-selective embolisation, resection

## Abstract

Arteriovenous malformations (AVMs) are uncommon errors of vascular morphogenesis; haemodynamically, they are high-flow lesions. Approximately 50% of AVMs are located in the craniofacial region. Subtotal excision or proximal ligation of the feeding vessel frequently results in rapid progression of the AVMs. Hence, the correct treatment consists of highly selective embolisation (super-selective) followed by complete resection 24–48 hours later. We treated 20 patients with facial arteriovenous malformation by using this method. Most of the lesions (80%) were located within the cheek and lip. There were no procedure related complications and cosmetic results were excellent.

## INTRODUCTION

Arteriovenous malformations (AVMs) are the result of errors of vascular development between the 4^th^ and 6^th^ weeks of gestation.[1] Failure to prune unwanted primitive communications between the arterial and venous systems may result in a malformation. Most of these lesions are obvious at birth while some are obvious during adolescence or adulthood. It is believed that enlargement is the result of changes in pressure and flow, ectasia, shunting and collateral proliferation, rather than cellular proliferation. AVMs have a tendency to grow with the child and after the individual has attained full growth, AVMs remain stable throughout life. Some enlargement may occur in response to trauma or hormonal disturbances during puberty or pregnancy.[2]

Treatment of AVMs can be difficult, as frequently, following an apparently successful extirpation, there is regrowth of the tumour to a size larger than its original size, often with supply by surgically inaccessible vessels.[3] Furthermore, the high flow rates and hyper vascularity of these lesions can lead to life-threatening complications, such as haemorrhage and/or cardiovascular instability. With the advent of super-selective angiography and new embolic agents, embolisation has become an integral part of treatment. The pre-operative embolisation diminishes blood loss and facilitates complete surgical extirpation. We report here our experience with super-selective embolisation, followed by surgical resection in 20 cases of facial AVMs.

## MATERIAL AND METHODS

Twenty consecutive patients with AVMs in the facial region were treated at the Army Hospital (R and R), Delhi Cantonment, in the period between December 2001 and October 2005. There were eight males and 12 females (male: female ratio of 1:1.5). Patients' age at presentation ranged from four to 56 years. Eighteen patients (90%) had a vascular malformation at birth or during infancy. In 16 (80%) patients, the majority of malformations occurred in the middle area of the face (cheek, nose, and upper lip). Two malformations (10%) occurred on the upper face (forehead) and two others (10%) on the lower face (lower lip) [Table 1]. The interval between the onset and presentation varied from a few days to 17 years, men tending to present later than women. Sixteen patients presented in clinical stage II and the remaining four in stage III of Schobinger's classification [Table 2]. The most common presenting features were facial disfigurement from the swelling, skin discoloration, and nasal bleeding. The case listed as serial No. 1 Table 1, Figure 1A presented with proptosis and severe epistaxis and required blood transfusion. All patients were evaluated preoperatively and detailed history was taken with regards to the onset and progression of the AVMs. The size and the site of the lesion was documented and associated symptoms and findings in the form of pain, ulceration, or bleeding were also noted. All AVMs were confirmed by colour Doppler and Magnetic Resonance Imaging (MRI) angiography [Figures 1B, 2B]. Computed tomography was done to exclude skeletal involvement in one patient who had a large orbital malformation. All AVMs were submitted to embolisation by the interventional radiologists. The embolic agents used were polyvinyl alcohol in 14 cases, absorbable gelatine sponge particles in four cases, and n-butyl cyanoacrylate (NBCA) in two cases. A super-selective embolisation was performed with the introduction of micro catheters into the distal feeding arteries as close to the nidus as possible. In the majority of cases, 90–100% devascularisation could be achieved and the lesions were resected 24–48 hours after the embolisation, except in one female patient (Case No: 19; Table 1) who had an AVM in her lower lip and was treated with NBCA and excision done 1 week later. In this patient due to extreme tortuosity of the feeder it became impossible for the interventional radiologist to negotiate a micro catheter. Since the lesion was superficial it could be embolised with direct percutaneous injection of NBCA under fluoroscopic control. Surgical resection was facilitated by the subcutaneous infiltration of adrenaline in normal saline (1: 2, 00,000). During resection, branches of the facial nerve were preserved [Figure 1D]. Four patients required resection of a portion of the involved skin along with resection of the malformation. Tissue deficits were covered with a split skin graft (on the forehead) and the rotation of a skin flap (on the cheek).

**Table 1:** Summary of treatment procedures

*Sr. No*	*Age (in years) / Sex*	*Location of AV Malformation*	*Clinical Stage*	*Arteries embolized*	*Interval between Embolisation and resection*	*Type of operative procedure*	*Outcome*
1.	56/M	Right orbit and right side of face	III	Right internal maxillary artery, right infra orbital artery, right lingual artery, right facial artery.	24 h	Excision of orbital lesion through medial orbitotomy, subcutaneous excision and sclerothrapy of facial component	No recurrence in 3& 1/2 yrs
2.	9/M	Right upper lip and check	II	Right superior labial artery and right facial artery	24 h	Subcutaneous extirpation	No recurrence in 14 months
3.	28/F	Right cheek, right nose, and right upper lip	II	Right Internal maxillary artery and right facial artery.	24 h	Subcutaneous extirpation	No recurrence in 5 months
4.	24/F	Left side of nose and Intra-nasally	III	Left facial artery and left internal maxillary artery	48 h	Subcutaneous extirpation	No recurrence in 1 year and 7 months
5.	6/F	Right cheek and right lower lip	II	Right internal maxillary artery and right facial artery	24 h	Subcutaneous extirpation	No recurrence in 1& 1/2 years
6.	41/M	Right forehead	II	Right superficial temporal artery	24 h	Subcutaneous extirpation	No recurrence in 6 months
7.	12/F	Left side of upper lip	II	Left internal maxillary artery and left facial artery	48 h	Subcutaneous extirpation	No recurrence in 2 years
8.	40/M	Upper lip and right naso- labial area	II	Right internal maxillary artery and right facial artery	24 h	Subcutaneous extirpation	No recurrence in 16 months
9.	35/F	Right upper lip	II	Right facial artery	24 h	Subcutaneous extirpation	No recurrence in 14 months
10.	44/F	Left cheek, left side nose, left upper lip	II	Left internal maxillary artery, left facial artery	24 h	Subcutaneous extirpation	No recurrence in 4 months
11.	52/M	Left orbit and left side of face	III	Left internal maxillary artery, left infra orbital artery and Left facial artery	48 h	Excision of orbital lesion through medial orbitotomy and excision of facial component	No recurrence in 2 years
12.	7/M	Left upper lip and check	II	Left superior labial artery and left facial artery	24 h	Subcutaneous extirpation	No recurrence in 12 months
13.	8/F	Left cheek and left lower lip	II	Left Internal maxillary artery and left facial artery.	24 h	Subcutaneous extirpation	No recurrence in 2 years
14.	38/M	Left cheek and left side nose	II	Left internal maxillary Artery and Left facial artery	24 h	Subcutaneous extirpation	No recurrence in 16 months
15.	42/F	Right side cheek, Right side nose and Right upper lip	II	Right internal maxillary artery and right facial artery	24 h	Subcutaneous extirpation	No recurrence in 6 months
16.	26/F	Left cheek, left nose and left upper lip	II	Left IMA and Left facial artery	29 h	Subcutaneous extirpation	No recurrence in 6 months
17.	34/F	Left side upper lip	II	Left facial artery	24 h	Subcutaneous extirpation	No recurrence in 11 months
18.	37/M	Left forehead	II	Left superficial temporal artery	24 h	Subcutaneous extirpation	No recurrence in 9 months
19.	20/F	Lower Lip	II	Bilateral labial arteries	7 days	Subcutaneous extirpation	No recurrence in 16 months
20.	10/F	Right upper lip	II	Left internal maxillary artery and left facial artery	48 h	Subcutaneous extirpation	No recurrence in 1.5 yrs

**Table 2:** Schobinger classification of arteriovenous malformation

*Stage*	*Features*
I.	Cutaneous blush / warmth
II.	Bruit, audible pulsations, expanding lesion
III.	Same as above with pain, ulceration, bleeding, infection
IV.	Same as above with Cardiac failure

**Figure 1A:**
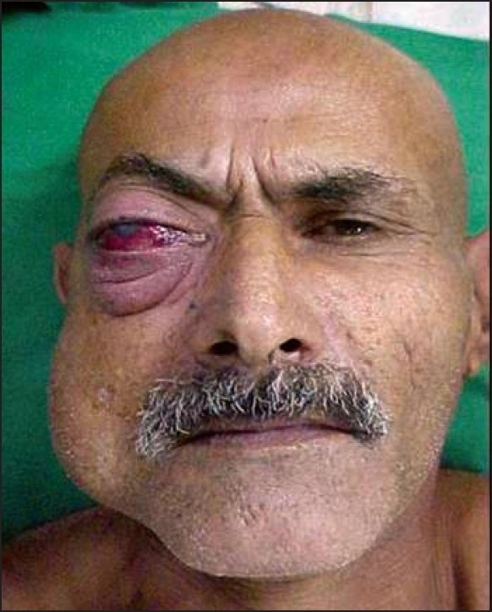
AVM involving the cheek and orbit, causing marked proptosis, chemosis, and poor vision

**Figure 1B:**
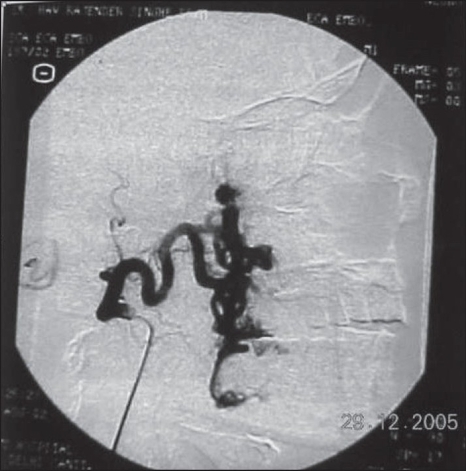
Angiogram revealed dilated anomalous vascular channels fed by internal maxillary artery, facial artery, ascending pharyngeal artery, infraorbital artery, nasal, and anterior ethmoidal arteries

**Figure 1C:**
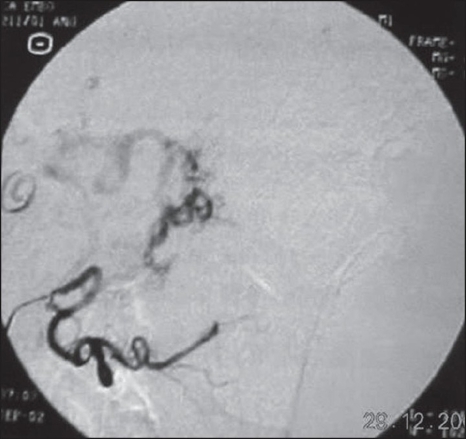
Post embolisation angiogram using PVA particles showed a marked reduction in vascularity

**Figure 1D:**
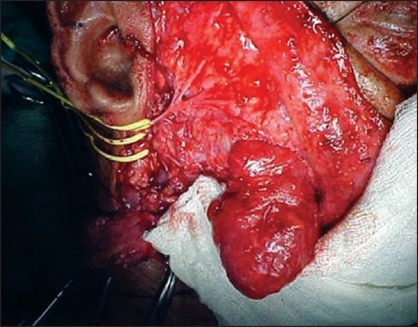
While dissecting the facial lesion, branches of facial nerve were safeguarded

**Figure 1E:**
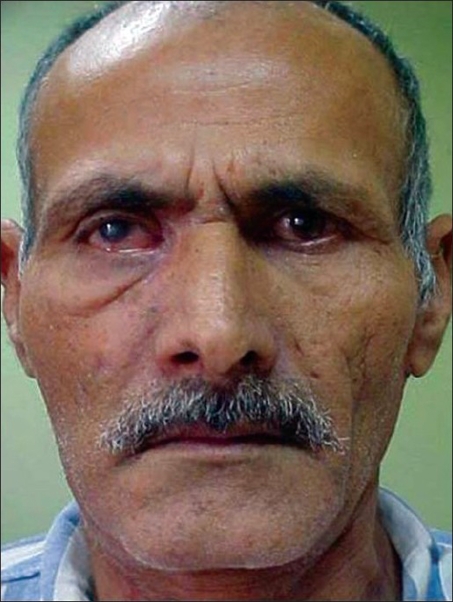
Postoperative result with improvement in vision

## RESULTS

Twenty consecutive patients with high-flow AVMs on the facial region were treated by super-selective embolisation, followed by surgical resection 24–48 h later except in one case where surgery was deferred by 7 days. Postembolisation arteriograms showed that 14 lesions were completely devascularised and five were effectively devascularised (> 90%). In one malformation (Case No: 3; Table 1), although only 70% devascularisation was achieved, the lesion was successfully resected with no significant blood loss. There were no embolisation-related complications with polyvinyl alcohol or gel foam particles. Two lip lesions treated with NBCA developed severe local reaction with edema and necrosis of tissues that healed well after debridement [Figures 2E and 2F]. In 18 out of 20 lesions, a complete resection was possible. In the patient with a combined orbital and facial malformation, a small portion of the lesion extended deep to the neck of mandible. After maximum possible surgical excision of this lesion, Sclerosant (Polidocanol) was injected into the inaccessible portion of the malformation.

**Figure 2A:**
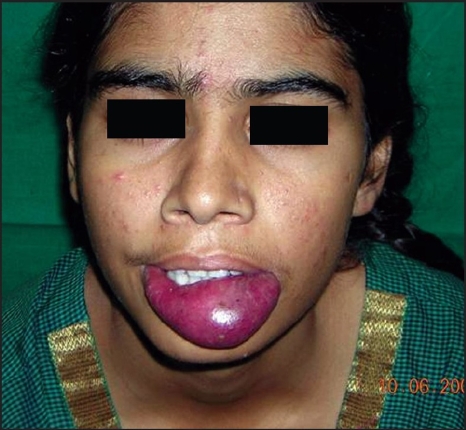
AVM lower lip

**Figure 2B:**
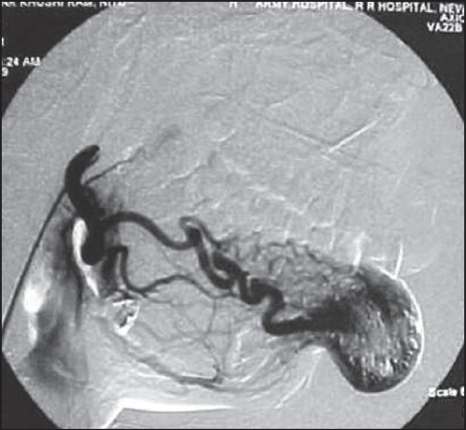
Angiogram revealed dilated anomalous vascular channels of both labial arteries

**Figure 2C:**
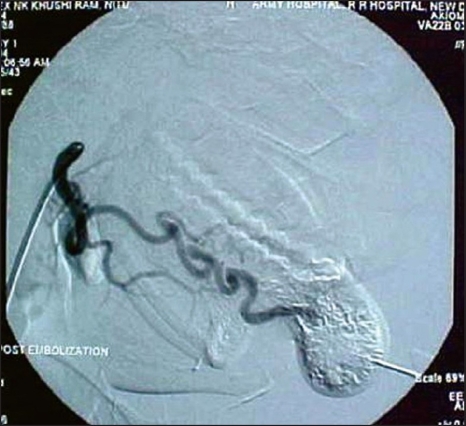
Angioembolization with NBCA

**Figure 2D:**
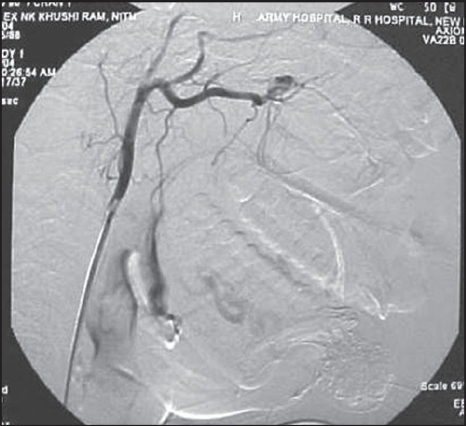
Postembolisation with NBCA

**Figure 2E:**
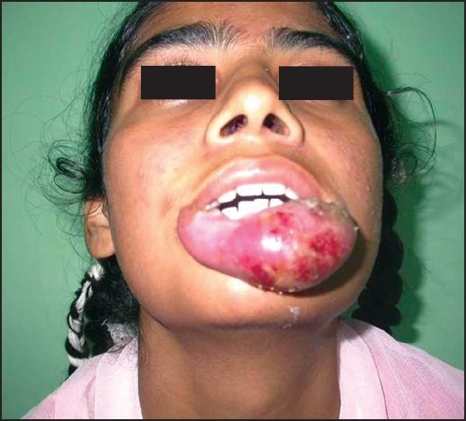
Local reaction with NBCA

**Figure 2F:**
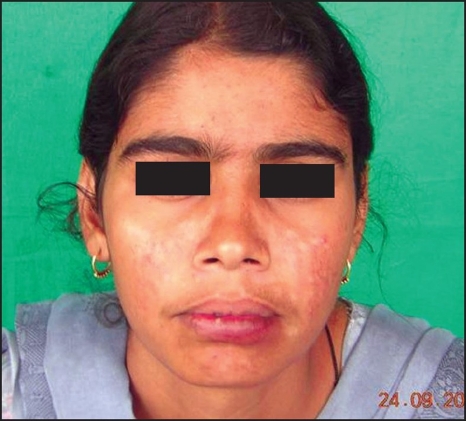
Postoperative appearance

Transient neuropraxia of the VII^th^ nerve occurred in one patient (Case No. 3) but improved in a week's time. Postoperative results were pleasing with improvement in both cosmesis and function. No recurrences were observed in the follow-up period which ranged from five months to three and half years (average period 15.2 months).

## DISCUSSION

AVMs are the result of a failure of regression of the arteriovenous channels in the primitive retiform plexus.[1] They are composed of a central nidus with anomalous congenital shunts between the arterial and venous systems. These abnormal vascular channels may not canalize or conduct blood flow for many years. An enlargement is the result of dilatation of the adjacent arteries and veins (collateralisation and recruitment)[4] rather than endothelial proliferation. Clinically, AVMs present with pain, hyperaemia, thrill, trophic changes, ulceration, and bleeding. Puberty and pregnancy affect the onset and progression of these lesions.

AVMs can be diagnosed with Pulsed Doppler which documents the arterial output and can be used to follow the progression of an AVM. With MRI best demonstrating the extent of malformation, angiography is unnecessary until intervention is contemplated.

AVMs present a therapeutic challenge because of their haemodynamic characteristics and their modality of growth. Surgical resection is often associated with extensive blood loss and an incomplete resection frequently leads to re-growth of the tumour to sizes that are often larger than its original size.[3] Proximal ligation of the parent vessel should be avoided as it is ineffective and may aggravate the problem making future endovascular therapy difficult or impossible.

In an effort to circumvent these problems, preoperative embolisation of the feeding arteries has been shown to reduce the hypervascularity and therefore, to aid surgical resection of these lesions. Recent developments in the design of micro catheters and distal navigation techniques have facilitated the catheterization of feeding arteries close to the nidus. The first therapeutic vascular embolisation using muscular tissue was described in 1930 by Brooks.[5] In 1972, Longacre *et al.*[6] reported good results with intravascular embolisation of a facial haemangioma using silicone balls impregnated with barium or tantalum. At present, the embolic materials generally employed are absorbable gelatine foam, polyvinyl alcohol (PVA), absolute alcohol, and NBCA. Gel foam is a temporary vessel occluder and will be resorbed in 1–2 weeks.[3] Polyvinyl alcohol is a permanent material and one of the most commonly used embolising agents. NBCA and alcohol are liquid embolising agents. NBCA has been used in the direct puncture technique in scalp arteriovenous malformations. Direct puncture with NBCA is an effective and safe technique for preoperative devascularisation of craniofacial AVMs.[7–9] Direct puncture embolisation of the venous pouch of an AVM has the advantages of reducing the risk of ischemia (i.e, skin necrosis and compromise of the central retinal artery) and being technically simple when compared to transarterial embolisation.[9] This embolising agent i.e. NBCA should be used with extreme care as being a liquid, it may progress more distally within the vasa nervosa leading to cranial nerve deficits.[10,11]

Complications from embolisation are infrequently seen; however, necrosis of adjacent tissues may occur. Hence, patients must be adequately treated with broad-spectrum antibiotics. The most serious complication is the backflow of the embolus into the internal carotid or vertebral arteries as a result of circulatory sluggishness. A distal placement of the micro catheter close to the nidus will avoid this complication.

Highly selective embolisation as a single treatment modality is rarely successful with high-flow lesions because of the later development of new vascular pathways.[12,13] However, it leads to a significant reduction in the blood flow within the vascular tumour which decreases operative blood loss and permits complete resection of the tumour.[14,15] Experience has shown that the 24 to 48 h period following embolisation is the ideal time for surgical intervention. The aim is complete resection, unlike staged resection that is applicable to slow-flow vascular malformations to minimize the chances of recurrence. The pattern of bleeding from the wound edges is the best way to determine whether or not the resection is adequate. A more than normal bleeding indicates presence of residual malformed tissue. Combined embolisation and resection is most successful for well-localized stage I or stage II AVMs.[16]

To conclude, super-selective embolisation followed by a complete surgical resection is the correct treatment for arteriovenous malformations on the facial region. Surgical resection of the vascular tumour should be performed with a skin-sparing incision to avoid disfigurement. Excision should be followed by primary closure whenever possible. Superficial tissue losses can be covered with a split thickness skin graft. Local tissue flaps (skin rotation flap for cheek) and Estlander's flap for the upper lip will give better cosmetic results. There may be a need for free tissue transfer in the reconstruction of more complex defects. After this combined modality of embolisation, followed by resection, the patient must be followed up for years by clinical examination, ultrasonography, and/or MRI.
